# KRAS^G12C^ Can Either Promote or Impair Cap-Dependent Translation in Two Different Lung Adenocarcinoma Cell Lines

**DOI:** 10.3390/ijms22042222

**Published:** 2021-02-23

**Authors:** George Kyriakopoulos, Vicky Katopodi, Ilias Skeparnias, Eleni G. Kaliatsi, Katerina Grafanaki, Constantinos Stathopoulos

**Affiliations:** 1 Department of Biochemistry, School of Medicine, University of Patras, 26504 Patras, Greece; g.kyriakopoulos@upnet.gr (G.K.); vasiliki.katopodi@kuleuven.be (V.K.); iskeparnias@upatras.gr (I.S.); e_kaliatsi@upatras.gr (E.G.K.); grafanaki@upatras.gr (K.G.); 2 Laboratory for RNA Cancer Biology, Department of Oncology, KU Leuven, 3001 Leuven, Belgium; 3 Department of Dermatology, School of Medicine, University of Patras, 26504 Patras, Greece

**Keywords:** KRAS^G12C^, signal transduction, translation initiation, lung cancer

## Abstract

KRAS^G12C^ is among the most common oncogenic mutations in lung adenocarcinoma and a promising target for treatment by small-molecule inhibitors. KRAS oncogenic signaling is responsible for modulation of tumor microenvironment, with translation factors being among the most prominent deregulated targets. In the present study, we used TALENs to edit EGFR^WT^ CL1-5 and A549 cells for integration of a Tet-inducible KRAS^G12C^ expression system. Subsequent analysis of both cell lines showed that cap-dependent translation was impaired in CL1-5 cells via involvement of mTORC2 and NF-κB. In contrast, in A549 cells, which additionally harbor the KRAS^G12S^ mutation, cap-dependent translation was favored via recruitment of mTORC1, c-MYC and the positive regulation of eIF4F complex. Downregulation of eIF1, eIF5 and eIF5B in the same cell line suggested a stringency loss of start codon selection during scanning of mRNAs. Puromycin staining and polysome profile analysis validated the enhanced translation rates in A549 cells and the impaired cap-dependent translation in CL1-5 cells. Interestingly, elevated translation rates were restored in CL1-5 cells after prolonged induction of KRAS^G12C^ through an mTORC1/p70S6K-independent way. Collectively, our results suggest that KRAS^G12C^ signaling differentially affects the regulation of the translational machinery. These differences could provide additional insights and facilitate current efforts to effectively target KRAS.

## 1. Introduction

In lung cancer, oncogenic mutations detected in *EGFR* (15–50%) or *KRAS* (20–30%) activate either MAPK and/or PI3K/AKT/mTOR signaling pathways, which, in turn, target important translation initiation factors [1]. KRAS is constitutively activated through mutations mainly at residues G12, G13 and Q61, leading to aberrant signal transduction, even in the absence of extracellular stimuli [2,3]. *KRAS* alleles have been associated with differences in the biochemical properties, structural conformation and biological activity of each mutant. These differences account for the different clinical phenotypes of KRAS-driven tumors, the prognostic value of the specific codon mutations and the response to first-line chemotherapy treatment, which vary between different cancer types and genomic contexts [4]. The most common mutation in lung adenocarcinoma is KRAS^G12C^ (~6.7%), which was recently successfully targeted by small-molecule inhibitors [5,6]. However, several previous and ongoing attempts to target KRAS-driven cancers have showed encouraging results but with high incidents of partial response, indicating that modulation of oncogenic KRAS signals is more complex than expected [7]. Translation of the genetic information is often deregulated in cancer due to oncogenic signaling and provides cancer cells with the advantage of increased expression of oncogenes or reduced expression of tumor suppressors [8]. Thus, modulation of MAPK and/or PI3K/AKT/mTOR signaling pathways allow cells to adapt their metabolism under stress conditions to promote cell survival and proliferation. Indeed, it was shown recently that mutant KRAS can rewire the metabolic process of cancer cells by transcriptionally activating NRF2, leading to acquired chemoresistance that can be effectively targeted [9]. KRAS-driven cell transformation is achieved through downstream targeting of important translation factors, mainly at the initiation level which is considered the rate-limiting step of the overall process [10]. In addition, the shift between cap-dependent and -independent translation under stress provides the necessary proteome changes that sustain metabolic rewiring and cancer progression. In the past years, a more detailed understanding of the translation regulation complexity has been under the spotlight of several reports highlighting the therapeutic potential of targeting the translational machinery of cancer cells [8,11]. However, in lung adenocarcinoma, a clear picture of the specific modulation of translation initiation driven by specific KRAS mutations is limited.

In the present study, we examined the effect of a tetracycline-inducible KRAS^G12C^ mutation in two different cell lines which do not harbor EGFR mutations (EGFR^WT^), thus excluding the possibility of EGFR-driven oncogenic signals. The inability of existing therapies based on either anti-EGFR monoclonal antibodies or small molecule inhibitors underlies the necessity of understanding KRAS intracellular signaling into more detail. We edited CL1-5 TP53^R248W^ cells via TALENs, which contain KRAS^WT^ and A549 TP53^WT^ cells, which, in addition, bear a KRAS^G12S^ mutation. In both cases, the effects of Tet-induced KRAS^G12C^ were compared to the corresponding control without addition of doxycycline, thus subtracting all the effects on translation initiation caused by factors others than KRAS^G12C^. Moreover, the fact that KRAS^G12C^ was further overexpressed intensifies the effects of the specific mutant. Indeed, induction of KRAS^G12C^ led to enhanced downstream signaling events in both cell lines, excluding the possibility of an antagonistic effect of the two KRAS mutants present in A549 cells. We measured, in comparison, the differential activation effects on both MAPK and PI3K/AKT/mTOR signaling pathways and observed that the major differences occur in the PI3K/AKT/mTOR cascade and especially in the mTOR complex formation, which has a nodal role as stress sensor. Similarly, we observed differences in translation initiation factors and an overall differential response that affects translation initiation between the two cell lines. Interestingly, puromycin labeling assays of nascent peptides synthesis and polysome profile analysis, showed expected upregulation of cap-dependent translation in A549 cells, but significant downregulation of translation rates in CL1-5 cells. The cancerous phenotype of CL1-5 cells relapsed after prolonged expression of KRAS^G12C^, which was accompanied by downregulation of p70S6K phosphorylation, suggesting possible mediation of cap-dependent translation by additional factors. One such mechanism was the downregulated phosphorylation of eIF2α, a key checkpoint of translation initiation regulation. Collectively, our results indicate that KRAS^G12C^ signaling has different effects on translation initiation of cancer cells with different oncogenic mutations, that could account for the partial inability of KRAS^G12C^-specific anticancer drugs to effectively slow down cancer progression [3].

## 2. Results and Discussion

### 2.1. KRAS^G12C^ Signaling Differentially Affects MAPK and PI3K/AKT/mTOR Pathways

**Figure 1:**
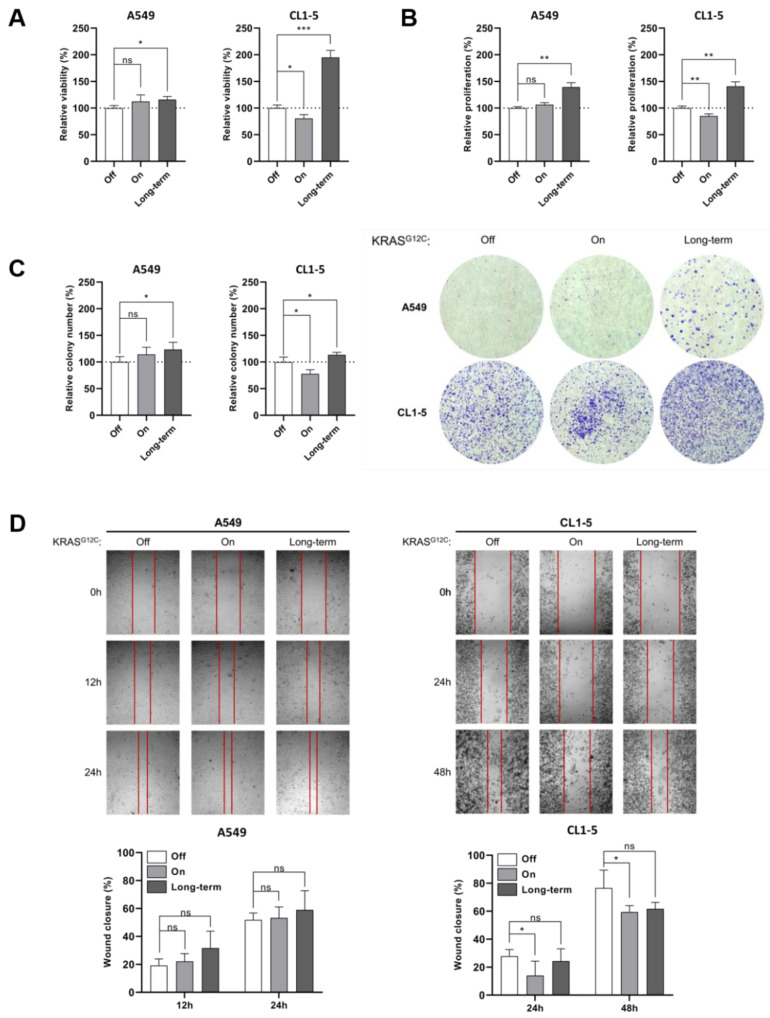
Phenotypic characterization of A549 and CL1-5 KRAS^G12C^ Tet-inducible stable cell lines. (**A**) Cell viability assay using ready resazurin solution and absorbance measurement at 570 nm and 600 nm before the addition of doxycycline (Off) and after short-term (72 h) and long-term (>1 month) induction of KRAS^G12C^ in A549 and CL1-5 cells. All levels are shown as a percentage (%) of relative viability as compared to the control (Off condition for both cases). (**B**) Cell proliferation assay using 0.2% crystal violet in 20% methanol and absorbance measurement at 590 nm before addition of doxycycline (Off) and after short-term (72 h) and long-term (>1 month) induction of KRAS^G12C^ in A549 and CL1-5 cells. All levels are shown as a percentage (%) of relative proliferation as compared to the control (Off condition for both cases). (**C**) Colony-formation assay before addition of doxycycline (Off) and after short-term (72 h) and long-term (>1 month) induction of KRAS^G12C^ in A549 and CL1-5 cells. All levels are shown as a percentage (%) of relative colony number as compared to the control (Off condition for both cases). (**D**) Wound healing assay before addition of doxycycline (Off) and after short-term (72 h) and long-term (>1 month) induction of KRAS^G12C^ in A549 at 12 h and 24 h and in CL1-5 cells at 24 h and 48 h following the wound procedure. Each time-point for each condition was compared to the initial wound at 0 h and the statistical analysis involved the comparison of On and long-term conditions with the Off condition. All levels are shown as a percentage (%) of wound closure as compared to the control (0 h). Data are compared by using unpaired Student’s *t*-test and are presented as means ± SD of biological triplicates. *P*-values are indicated with * *p* < 0.05; ** *p* < 0.01; *** *p* < 0.001.

We constructed A549 and CL1-5 stable cell lines with an integrated Tet-inducible KRAS^G12C^ expression system. The construct containing the *KRAS4B* c.34G>T gene with an upstream Tet-promoter was integrated into the AAVS1 locus of both cell lines using TALE Nucleases to insert double strand breaks and promote integration of the construct with homologous recombination, as previously described [12]. After confirming the successful integration of the construct and the expression of KRAS^G12C^ with the addition of doxycycline in both cell lines, we phenotypically characterized the cells (Figure 1A–D and Supplementary Figure S1A–B). 

**Figure 2:**
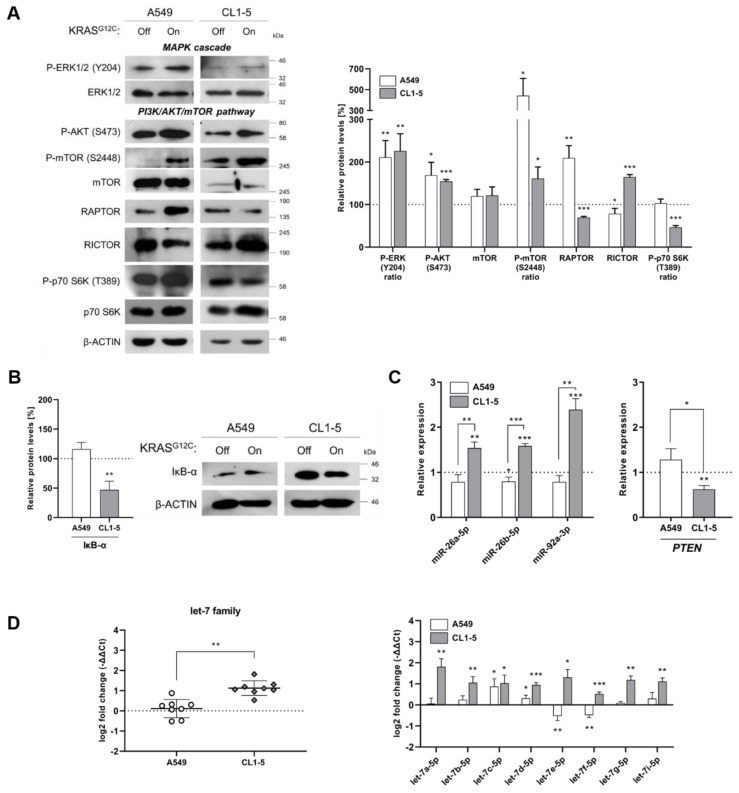
KRAS^G12C^ differentially affects MAPK and PI3K/AKT/mTOR pathways effectors. (**A**) Immunoblot analysis of MAPK and PI3K/AKT/mTOR signaling effectors in A549 and CL1-5 cells before (Off) and after (On) KRAS^G12C^ short-term induction with doxycycline. The protein levels were normalized against *β*-actin levels in each separate set of experiments. Band intensities are shown as a percentage (%) of relative protein levels as compared to the control (dotted line). Phosphorylation levels are shown as a ratio of phosphorylated to total relative protein levels, where indicated. (**B**) Immunoblot analysis of the IκB-α inhibitor of NF-κB in A549 and CL1-5 cells before (Off) and after (On) KRAS^G12C^ short-term induction with doxycycline. The protein levels were normalized against *β*-actin levels in each separate set of experiments. Band intensities are shown as a percentage (%) of relative protein levels as compared to the control (dotted line). (**C**) Expression levels of miRNAs and *PTEN* gene as measured by RT-qPCR in A549 and CL1-5 cells before (Off) and after (On) KRAS^G12C^ short-term induction with doxycycline. All levels are shown as relative expression compared to the control (dotted line) using the 2^-ΔΔCt^ method. (**D**) Cumulative expression profile of all members of the let-7 family of miRNAs in A549 and CL1-5 cells before (Off) and after (On) KRAS^G12C^ short-term induction with doxycycline (left panel). Expression levels of each member are also shown (right panel). All levels are shown as log2 fold change compared to the control (corresponding to 0 log2fold) using the -ΔΔCt method. Data are compared by using unpaired Student’s *t*-test and are presented as means ± SD of biological triplicates. P-values are indicated with * *p* < 0.05; ** *p* < 0.01; *** *p* < 0.001.

A549 cells showed slightly elevated cell proliferation after induction of KRAS^G12C^ compared to the untreated cells and metastatic potential with no statistical significance. On the other hand, the proliferation and metastatic potential of CL1-5 cells were downregulated after short-term KRAS^G12C^ induction, and interestingly, were restored and upregulated further after prolonged expression of KRAS^G12C^ (Figure 1A–D). It should be noted that apoptosis is possibly not induced, as shown by the expression levels of BCL2 family and mitochondrial ROS production (Supplementary Figure S2A–B). We hypothesized that KRAS^G12C^ downstream signaling could differ in the two cell lines, thus possibly leading to differential targeting of translation. Further analysis showed that KRAS^G12C^ activated the MAPK and PI3K/AKT pathways in both cell lines. However, although in both cases mTOR was activated through phosphorylation, we observed an interesting and significant difference in the phosphorylation level between the two cell lines, which pointed to possible differences in the activation of the two mTOR complexes (Figure 2A). After measuring the expression of RAPTOR and RICTOR, which are the scaffold proteins for the formation of mTORC1 and C2 complexes, respectively, we observed that in A549 cells, RAPTOR was upregulated, but RICTOR was slightly downregulated, while in CL1-5 cells, the expression pattern was inverted (Figure 2A). This observation suggests that KRAS^G12C^ expression in the two cell lines could result in selective modulation of the mTORC1 and C2 complex formation, with possible similar effects on downstream targeting. Impairment of mTORC1 complex formation in CL1-5 cells is further supported by the observation of p70S6K hypophosphorylation, which is a substrate for mTORC1. The same phosphorylation in A549 cells remained in basal levels (Figure 2A). Accordingly, the preference of signal transduction through mTORC2 complex formation in CL1-5 cells was correlated with downregulation of IκB-α, a major inhibitor of NF-κB, leading to activation of NF-κB and upregulation of *RICTOR* (Figure 2B and Supplementary Figure S3). It has been proposed that NF-κB can be activated through the mTORC2 complex, which in turn upregulates *RICTOR* levels. Of note, the mTORC2/NF-κB axis has been correlated with resistance to anticancer agents and the canonical NF-κB pathway differentially affects ROS-induced DNA damage response in normal and tumor lung cells [13,14,15]. mTORC1 has been extensively studied as a key modulator of cell proliferation and metabolic changes through various downstream effectors, with p70S6K and 4E-BP1 being the best-characterized targets. However, far less is known about the mTORC2 complex, which promotes cell survival through the phosphorylation of AKT at residue Ser473. Although both complexes are targeted by the highly specific mTOR inhibitor rapamycin, mTORC2 is relatively insensitive to the drug at nanomolar concentrations [16]. This further supports the notion that the two complexes are controlled by different cellular networks, in which KRAS mutants could be major contributors. An interesting finding is the role of the AMP-activated protein kinase (AMPK) and phospholipase D (PLD) on mTOR targeting. AMPK was found to suppress PLD, leading to lower levels of phosphatidic acid (PA), a metabolite that antagonizes the inhibitory effects of rapamycin and has differential affinity to mTORC1 and mTORC2 [17,18]. Since AMPK can impair mTORC1 signaling and has been linked to KRAS driven tumors, the possible participation of AMPK and PLD to differential mTOR complex formation by KRAS^G12C^ requires further elucidation [19,20].

In addition, we looked for differences at the post-transcriptional level and we measured the expression of miR-26a-5p, miR-26b-5p and miR-92a-3p, which downregulate *PTEN*, a known tumor-suppressor and major regulator of the PI3K/AKT pathway [21]. We observed that KRAS^G12C^ induction upregulated the expression of all three miRNAs only in CL1-5 cells (Figure 2C). Moreover, we observed upregulation of all members of the tumor suppressor let-7 family, again only in CL1-5 cells (Figure 2D). The let-7 family of miRNAs targets major oncogenes, including *KRAS* and *c-MYC*, and upregulation of specific members of the let-7 family has been detected in senescent human lung fibroblasts [22,23]. Therefore, our observation suggests induction of a protective response against the oncogenic signal of KRAS^G12C^ in CL1-5 cells. Taken together, our results indicate that KRAS^G12C^ activates equally the MAPK pathway in both cell lines. In A549 cells, the PI3K/AKT signal is transduced possibly through the activation of the mTORC1 axis, which presumably leads to the stimulation of translation initiation. On the other hand, in CL1-5 cells the mTORC1/p70S6K is downregulated and, instead, the formation of mTORC2 complex is favored. The latter is currently considered a novel KRAS direct effector activator of NF-κB [14,24].

### 2.2. Reduced Fidelity during mRNA Scanning and Start Codon Selection

**Figure 3:**
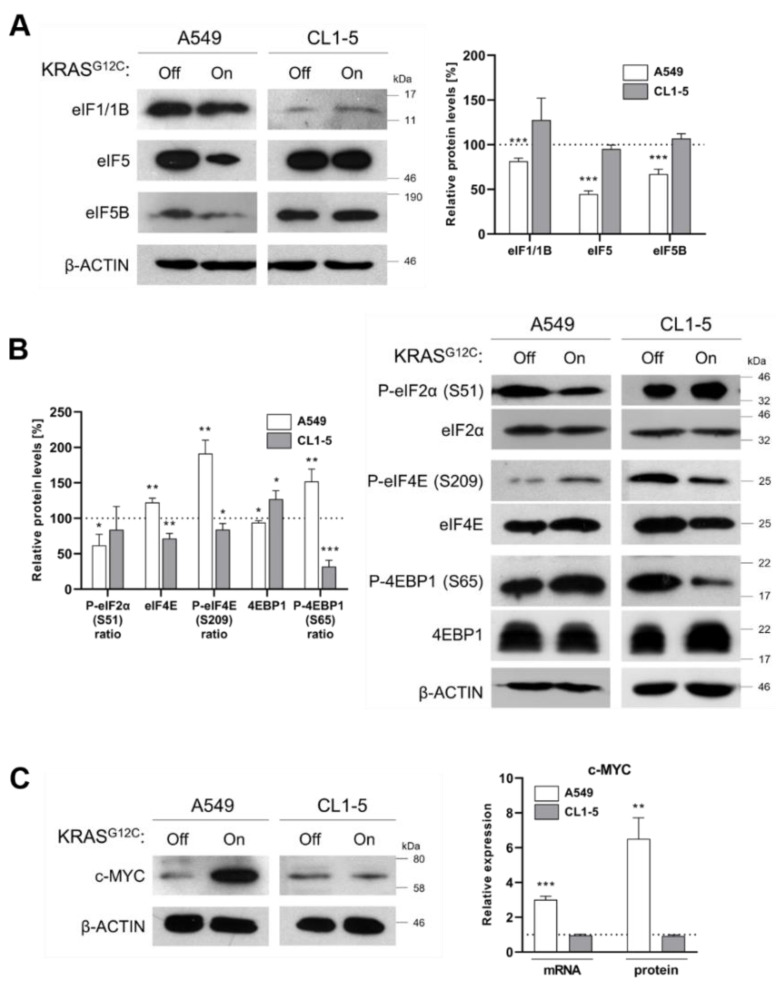
KRAS^G12C^ affects translation fidelity during mRNA scanning and cap-dependent translation initiation. (**A**) Immunoblot analysis of key translation initiation factors involved in the stringency of start codon selection during mRNA scanning in A549 and CL1-5 cells before (Off) and after (On) KRAS^G12C^ short-term induction with doxycycline. The protein levels were normalized against *β*-actin levels in each separate set of experiments. Band intensities are shown as a percentage (%) of relative protein levels as compared to the control (dotted line). (**B**) Immunoblot analysis of key translation initiation factors that regulate global translation rates in A549 and CL1-5 cells before (Off) and after (On) KRAS^G12C^ short-term induction with doxycycline. The protein levels were normalized against *β*-actin levels in each separate set of experiments. Band intensities are shown as a percentage (%) of relative protein levels as compared to the control (dotted line). Phosphorylation levels are shown as a ratio of phosphorylated to total relative protein levels. (**C**) Immunoblot analysis of c-MYC in A549 and CL1-5 cells before (Off) and after (On) KRAS^G12C^ short-term induction with doxycycline. mRNA levels were identified by RT-qPCR and the relative protein levels were calculated after normalization against *β*-actin levels. Relative levels are shown as compared to the control (dotted line). Data are compared by using unpaired Student’s *t*-test and are presented as means ± SD of biological triplicates. *P*-values are indicated with * *p* < 0.05; ** *p* < 0.01; *** *p* < 0.001.

Scanning of mRNA and start codon selection is essential for translation initiation and predominantly involve eIF1 and eIF5 initiation factors [25]. Knockdown and knockout studies have been correlated to the depletion or deletion of eIF1 with loss of translation fidelity, increased rates of translation initiation of upstream ORFs (leaky scanning) and/or re-initiation of translation, mechanisms that favor cancer progression and result in cells with error prone translation machinery. In the same studies, eIF5 was also found downregulated in correlation to eIF1 expression pattern [26]. In our study, induction of KRAS^G12C^ led to the downregulation of eIF1/1B only in A549 cells (Figure 3A). Additionally, in agreement with previous reports, we found that eIF5 was downregulated in A549 cells, but not in CL1-5 cells (Figure 3A). It is known that eIF5 acts as GTPase-activating protein for eIF2 GTPase upon recognition of the initiator codon. Interestingly, eIF5 is required for selection of the appropriate start codon among adjacent AUGs and its downregulation in A549 cells could be also indicative of leaky scanning [27]. In addition, eIF5B was downregulated only in A549 cells, an observation that can be linked to the fact that in cases of cellular stress, eIF5B can substitute eIF2 in delivering the Met-tRNA_i_ and can stimulate a more efficient initiation codon recognition during mRNA scanning (Figure 3A) [28]. In conclusion, the downregulation of eIF1/1B, eIF5 and eIF5B that we observed in our study indicates a loss of stringency of start codon selection in A549 cells, a deregulation which is related to increased translation errors, which fuel tumorigenesis [29]. Interestingly, in CL1-5 cells, all these factors remained unaffected, an observation which implies that translation initiation is not the primary target of KRAS^G12C^ signaling and may be directly transduced through NF-κB activation (Figure 3A and Supplementary Figure S4A).

### 2.3. KRAS^G12C^ Modulates Cap-Dependent Translation Initiation

Phosphorylation of eIF2 and availability of eIF4E for pre-initiation complex formation, represent the two main checkpoints of translation initiation regulation [30]. The eIF2α subunit of the heterotrimeric eIF2 complex is regulated by the opposing effects of stress-sensing eIF2α kinases (GCN2, PERK, PKR) and the eIF2α phosphatase GADD34. Phosphorylation at serine 51 leads to inhibition of global translation by prevention of eIF2B-mediated recycling of GTP [31]. In our study, induction of KRAS^G12C^ in A549 cells led to lower phosphorylation ratio of eIF2α, indicating enhanced translation rates. Of note, in CL1-5 cells we did not observe alterations in the phosphorylation ratio of eIF2α (Figure 3B). In addition, in A549 cells eIF4E was overexpressed, accompanied by higher phosphorylation rates upon induction of KRAS^G12C^. This finding suggests promotion of cap-dependent translation, since recognition of the 5’ cap by eIF4E is considered the rate limiting step of translation [11]. On the other hand, in CL1-5 cells eIF4E was found both downregulated and hypophosphorylated, a pattern which suggests impaired cap-dependent translation (Figure 3B). The availability of eIF4E to interact with the 5’ cap of mRNAs in A549 cells is further supported by the observed elevated phosphorylation of 4E-BP1 at serine 65, which is known to prevent association of 4E-BP1 with eIF4E (Figure 3B) [32]. In CL1-5 cells, however, we observed very low phosphorylation levels of 4E-BP1 accompanied by upregulation of total 4E-BP1. These findings are consistent with the negative regulation of eIF4E by 4E-BPs and suggest lower translation rates (Figure 3B). The results are also in agreement with the observation of differential signaling that we described above. Positive regulation of eIF4E in A549 cells has been correlated to enhanced translation of subsets of mRNAs, termed “eIF4E-sensitive mRNAs,” which play a key role in proliferation, similar to c-MYC, as we also confirmed in A549 cells, but not in CL1-5 cells (Figure 3C) [11]. It has been reported that c-MYC induces a feedforward loop by enhancing the transcription of its own gene along with the eIF4F complex genes, leading to enhanced cap-dependent translation, which favors c-MYC translation [33]. Therefore, the expression profile of c-MYC can be considered an indirect indicator of cap-dependent translation. In the same line, in A549 cells we observed an upregulation of *eIF4E*, *eIF4AI* and *eIF4GI*, possibly due to upregulation of c-MYC (Supplementary Figure S4B). 

**Figure 4:**
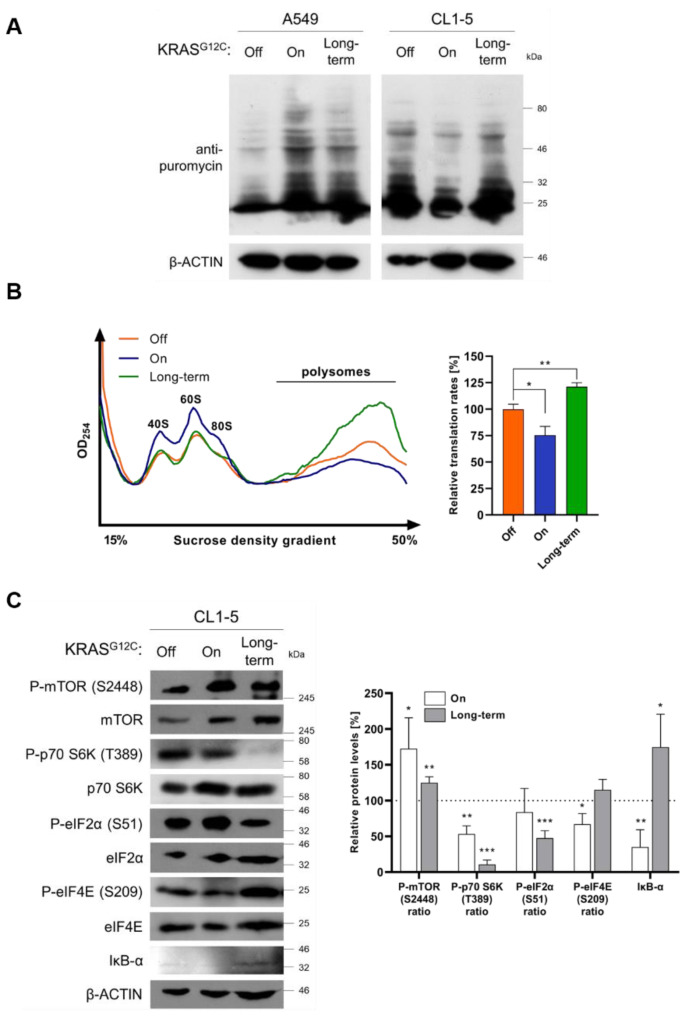
KRAS^G12C^ can either enhance or impair cap-dependent translation initiation. (**A**) Non-radioactive measurement of translation rates using puromycin staining of nascent peptides before addition of doxycycline (Off) and after short-term (72 h) and long-term (>1 month) induction of KRAS^G12C^ in A549 and CL1-5 cells. (**B**) Polysome profile analysis before addition of doxycycline (Off) and after short-term (72 h) and long-term (>1 month) induction of KRAS^G12C^ in CL1-5 cells analyzed on 15–50% sucrose density gradients upon treatment with cycloheximide. The peaks corresponding to ribosomal subunits (40S, 60S and 80S) and the polysomes tail are indicated. Relative polysome levels (%) were calculated by measuring the AUC on ImageJ (Fiji) and compared to the means AUC of the control triplicates. (**C**) Immunoblot analysis of key proteins involved in translation regulation in CL1-5 cells before addition of doxycycline (Off) and after short-term (72 h) and long-term (>1 month) induction of KRAS^G12C^ in CL1-5 cells. The protein levels were normalized against *β*-actin levels in each separate set of experiments. Band intensities are shown as a percentage (%) of relative protein levels as compared to the means values of the control triplicates. Phosphorylation levels are shown as a ratio of phosphorylated to total relative protein levels, where indicated. Data are compared by using unpaired Student’s *t*-test and are presented as means ± SD of biological triplicates. *P*-values are indicated with * *p* < 0.05; ** *p* < 0.01; *** *p* < 0.001.

To measure global translation on both cell lines, we performed puromycin staining after short (72 h) and long-term (>1 month) induction of KRAS^G12C^. As expected, we observed increased global translation in A549 cells during short-term and long-term induction (Figure 4A). However, in CL1-5 cells, during short-term KRAS^G12C^ induction, global translation was suppressed, a finding that indicates impairment of cap-dependent translation under these conditions [11]. Interestingly, when induction of KRAS^G12C^ was prolonged for over a month, translation was restored to basal levels (Figure 4A). Subsequent polysome profile analysis of CL1-5 cells verified the downregulation of translation rates based on the reduction of polysomes after short-term induction (Figure 4B blue line) and was followed by a significant increase of polysomes formation after long-term induction of KRAS^G12C^ (Figure 4B green line). Interestingly, this increase was not followed by elevated phosphorylation of p70S6K, but rather by further phosphorylation decrease, an unexpected finding which consolidates our previous observations that translation is not the predominant target of KRAS^G12C^ in CL1-5 cells (Figure 4C). However, cap-dependent translation was restored to basal levels after prolonged KRAS^G12C^ induction, as indicated by the expression level and phosphorylation of eIF4E. Interestingly, the observed stimulation of translation was accompanied by hypophosphorylation of eIF2α subunit of the ternary complex, a finding correlated with enhanced translation rates [31]. Finally, NF-κB was ultimately inhibited, since IκB-α was upregulated (Figure 4C).

**Figure 5:**
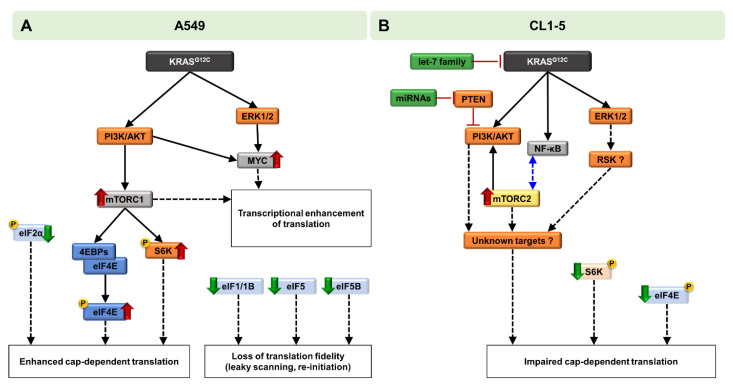
Schematic representation of the differential KRAS^G12C^-mediated oncogenic signaling and modulation of translation. (**A**) In A549 cells, expression of KRAS^G12C^ mediates its oncogenic signaling through the MAPK and PI3K/AKT/mTORC1/p70S6K signaling pathways. Downstream targeting of 4E-BPs and eIF4E, along with the hypophosphorylation of the eIF2α subunit, leads to enhanced cap-dependent translation. Downregulation of eIF1, eIF5 and eIF5B is possibly associated with loss of translation fidelity during scanning of the mRNA, while recruitment of c-MYC, along with activated mTORC1, further promotes translation at the transcription level. All these events lead to promotion of translation and enhanced cell proliferation. (**B**) In CL1-5 cells, expression of KRAS^G12C^ leads to activation of both MAPK and PI3K/AKT signaling pathways. PI3K axis activation is further enhanced by targeting of tumor-suppressor *PTEN* by miRNAs, while the tumor-suppressor let-7 family of miRNAs is upregulated due to a possible stress response mechanism. Interestingly, mTORC2 formation is promoted and is possibly correlated with the activation of NF-κB. Downstream transcriptional and translational events, with participation of unidentified effectors, which could involve the ERK1/2-targeted RSK (Ribosomal S6 Kinase), along with the hypophosphorylation of both p70S6K and eIF4E, lead to impairment of cap-dependent translation.

Our results suggest that the net effect of KRAS^G12C^ expression in A549 cells is the promotion of cap-dependent translation, which is in good agreement with previous reports suggesting that KRAS mutations can promote cap-dependent translation (Figure 5A) [34,35]. On the contrary, in CL1-5 cells, KRAS^G12C^ induces a stress response mechanism which initially inhibits cap-dependent translation, possibly to facilitate transcriptional and translational rewiring programs predominantly through the activation of NF-κB and the recruitment of unidentified downstream effectors recruited by AKT, ERK1/2 and possibly the ERK1/2-targeted RSK kinase [36]. The possibility of IRES-mediated translation of genes, although it was not tested in the present study, cannot be excluded for a subset of mRNAs (Figure 5B). It should be noted that these findings are compatible with the phenotype of both cell lines, after either short-term or prolonged induction of KRAS^G12C^ (Figure 1A–D).

## 3. Materials and Methods

### 3.1. Cell Lines and Generation of Tet-Inducible KRAS^G12C^ Expression System

A549 human lung adenocarcinoma cells were purchased from ATCC and CL1-5 human lung adenocarcinoma cells were kindly provided by Dr I. Habeos (University of Patras, School of Medicine). The full-length cDNA containing the KRAS c.34G>T (KRAS^G12C^) gene sequence was constructed into the pAAVS1-NDi-CRISPRi (Gen2) plasmid using the PacI and AgeI restriction sites to remove the CRISPRi gene and replace it with the KRAS^G12C^ gene sequence. pAAVS1-NDi-CRISPRi (Gen1) was a gift from Bruce Conklin (Addgene plasmid #73497; http://n2t.net/addgene:73497 (accessed on 22 February 2021)). Lipofectamine 2000 (Invitrogen) was used to transfect the cells with the plasmids containing the KRAS mutant and the two TALE Nucleases as previously described [12]. AAVS1-TALEN-L and AAVS1-TALEN-R were a gift from Danwei Huangfu (Addgene plasmid #59025; http://n2t.net/addgene:59025 (accessed on 22 February 2021) and Addgene plasmid #59026; http://n2t.net/addgene:59026 (accessed on 22 February 2021)). The pAAVS1-KRAS^G12C^ construct was integrated into the AAVS1 locus of A549 and CL1-5 cells using a TALEN-assisted gene-trap approach [12]. The TALENs produce double strand brakes on a specific region of the AAVS1 locus and the pAAVS1-KRAS^G12C^ construct is inserted with homologous recombination. Geneticin Selective Antibiotic (G418 Sulfate) (800 μg ml^-1^) (Gibco) was added into the cell cultures to select the cell clones stably transfected with the pAAVS1-KRAS^G12C^-TetOn construct and clonal selection by serial dilution in 96-well plates was performed. Clonal colonies were cultured and gDNA isolation was performed using TRIzol Reagent (Invitrogen), to confirm the integration of the construct to the specific locus by PCR with DreamTaq DNA polymerase (Thermo Scientific) using a set of primers that amplified the expected 1033 bps PCR product only if the construct was integrated into the AAVS1 locus (F: 5’-CCTCTAACGCTGCCGTCTCT-3’ and R: 5’-CTCCACGTCACCGCATGTTAG-3’). A549_TetOn_KRAS^G12C^ and CL1-5_TetOn_KRAS^G12C^ cell lines were routinely maintained in Dulbecco’s modified Eagle’s medium (DMEM) supplemented with 10% Fetal Bovine Serum (FBS), at 37 °C in a 5% CO_2_. KRAS^G12C^ expression was induced by 150 ng ml^-1^ doxycycline (Cayman) and confirmed by qRT-PCR. Long-term induction of KRAS^G12C^ was performed by culturing the cells with 150 ng ml^-1^ doxycycline for over 1 month (passages 16–20). For all experiments involving long-term induction (>1 month), the same passage of cells (P16–20) was also used for the short-term condition and both were compared to the same control. For all other experiments, passages 8–12 were used. Low levels of doxycycline were used to induce a moderate upregulation of KRAS^G12C^, as has also been described elsewhere [37].

### 3.2. Cell Viability Assay

Five thousand cells were seeded in 96-well plates in DMEM/10% FBS and were treated with 150 ng ml^-1^ for short-term (72 h) and long-term (>1 month) KRAS^G12C^ induction. Cells were incubated for 2 h with ready resazurin solution (Biotium) at 37 °C followed by absorbance-based detection (570 nm and 600 nm) according to the manufacturer’s instructions. Experiments were performed in triplicates.

### 3.3. Cell Proliferation Assay

Fifty thousand cells were seeded in six-well plates in DMEM/10% FBS and were treated with 150 ng ml^-1^ for short-term (72 h) and long-term (>1 month) KRAS^G12C^ induction. Cells were washed twice with 1× PBS and stained with 1 mL 0.2% crystal violet (Sigma-Aldrich, St. Louis, MO, USA) in 20% methanol for 10 min. Cells were then washed three times with H_2_O and were then solubilized with 0.5 mL 10% acetic acid for 20 min. Solubilized crystal violet was measured at 590 nm according to the manufacturer’s instructions. Experiments were performed in triplicates.

### 3.4. Colony-Formation Assay

Five thousand cells were seeded in 48-well plates in DMEM/10% FBS and were treated with 150 ng ml^-1^ for short-term (72 h) and long-term (>1 month) KRAS^G12C^ induction. Cells were then trypsinized, diluted and seeded in 100 mm dishes until colonies became visible. Cells were then stained with 0.2% crystal violet (Sigma-Aldrich, St. Louis, MO, USA) in 20% methanol for 10 min and were then washed three times with Η_2_Ο. Images were captured, and colonies were quantified using ImageJ (Fiji, ImageJ, Wayne Rasband National Institutes of Health). Experiments were performed in triplicates.

### 3.5. Wound-Healing Assay

Cells were seeded in 12-well plates and were treated with 150 ng ml^-1^ for short-term (72 h) and long-term (>1 month) KRAS^G12C^ induction until they reached monolayer confluency. Wounds were made by scratching the monolayer using a 100 μL pipette tip. Images were captured following the wound procedure and at multiple time points (12 h, 24 h and 48 h) and the scratched area was quantified at each time point using ImageJ plugin MRI Wound Healing tool (Volker Baecker, Montpellier RIO Imaging, Montpellier, France). Experiments were performed in triplicates.

### 3.6. Immunoblotting and Non-Radioactive Measurement of Translation Rates

Whole cell lysate was extracted with lysis buffer (20 mM Tris-HCl pH 7.4, 120 mM KCl, 1 mM EDTA, 1 mM DTT, 2% NP-40, 1% (*v*/*v*) Protease Inhibitor Cocktail (Sigma-Aldrich, St. Louis, MO, USA) and 1 mM Na_3_VO_4_). Of the total protein extracts, 30–50 μg was separated by SDS-PAGE and transferred to Immobilon-P PVDF membranes (Millipore, Massachusetts, MA, USA). Blocking in 5% (*w/v*) non-fat dry milk in TBS/0.05% Tween 20 was followed by incubation with primary antibodies overnight at 4 °C and with goat anti-mouse or anti-rabbit secondary HRP-conjugated antibodies for 1 h at room temperature. The band intensity measurements of each experiment were analyzed using the Image Lab software (Bio-Rad, version 6.1, Berkeley, CA, USA). The protein levels were normalized against *β*-actin levels in each separate set of experiments. For non-radioactive measurement of translation rates, 1 μM puromycin was added to the culture medium for 10 min and the levels of puromycylated nascent peptides were assessed by immunoblotting. Puromycylated peptides levels have been established to be proportional to global translation [38]. A complete list of the antibodies used can be found in Supplementary Table S1.

### 3.7. Real-Time Quantitative PCR

Cells (0.3 × 10^6^ cells) were immediately lysed using TRIzol Reagent (Invitrogen, Waltham, MA, USA) to isolate RNA fractions. For small RNA isolation (<200 nt), part of the same sample was further treated using mirVana Isolation Kit (Invitrogen, Waltham, MA, USA) following the manufacturer’s instructions. In both cases, RNA was treated with DNase I (New England Biolabs, Ipswich, MA, USA). The quantity and yield of RNA was evaluated by Multiskan Sky Microplate Spectrophotometer. Long RNA fraction (>200 nt) was reverse transcribed using SuperScript II (Invitrogen, Waltham, MA, USA) and Random Hexamers primer according to the manufacturer’s protocol. Small RNA fraction (<200 nt) was first polyadenylated using *E. coli* Poly(A) Polymerase (New England Biolabs, Ipswich, MA, USA) and reverse transcribed using SuperScript II (Invitrogen, Waltham, MA, USA) and oligodT adapter primer, which contains the sequence recognized by the reverse primer (outer primer) for detection of miRNAs. qPCR reactions were performed using KAPA SYBR FAST qPCR Kit (Kapabiosystems, Cape Town, Western Cape, South Africa) using 50 ng cDNA as template. Reactions were set up in 96-well plates and performed on an MX3000P qPCR system (Agilent, Santa Clara, CA, USA). The Ct values were analyzed using the 2^-ΔΔCT^ method after normalization against *ACTB* levels for genes and miR-103 for miRNAs [39]. All reactions were performed in triplicates and the primers sequences are shown in Supplementary Tables S2 and S3.

### 3.8. Polysome Profile Analysis

Cells were incubated at 37 °C with 100 μg ml^-1^ cycloheximide (CHX) for 10 min and then washed with PBS/100 μg ml^-1^ CHX, scraped and lysed into 1 mL of lysis buffer (20 mM Tris-HCl pH 7.4, 150 mM KCl, 7 mM MgCl_2_, 1 mM DTT, 1% NP-40, 1% (*v/v*) Protease Inhibitor Cocktail (Sigma-Aldrich, St. Louis, MO, USA) 40U ml^-1^ RNase Inhibitor Murine (New England Biolabs, Ipswich, MA, USA) and 100 μg ml^-1^ CHX). Then, 20 OD_260_ units were loaded onto a 15–50% sucrose density gradient, centrifuged in an SW41 rotor at 39,000 rpm for 2 h and 30 min at 4 °C and analyzed by optical scanning at 254 nm.

### 3.9. Measurement of Mitochondrial ROS Production Using Flow Cytometry

Cells were seeded in six-well plates and were treated with 150 ng ml^-1^ for short-term (72 h) and long-term (>1 month) KRAS^G12C^ induction. Then, 0.5 × 10^6^ cells were stained using 2.5 µM MitoSOX Red dye (Invitrogen, Waltham, MA, USA) for 30 min at 37 °C. Subsequently, the cells were washed gently three times with 37 °C-prewarmed complete PBS (CPBS). The stained cells were analyzed using a BD FACS Calibur. The results are expressed as a histogram presenting cell fluorescence emission and were quantified using the mean fluorescence intensity (MFI).

### 3.10. Statistical Analysis

All data are presented as relative expression levels compared to the mean levels of control replicates. Statistical analysis was performed using GraphPad Prism v.8.0.2 (GraphPad Software Inc., San Diego, CA, USA). Data were compared using unpaired Student’s *t*-test and are presented as means ± SD of the biological triplicates. Details are indicated in the respective figure legends.

## 4. Conclusions

In conclusion, induction of KRAS^G12C^ in A549 cells targets translation initiation and leads cells to elevated global translation rates through activation of the mTORC1 axis. In CL1-5 cells, the short-term induction leads to impairment of cap-dependent translation and a possible switch to IRES-mediated protein synthesis. However, prolonged expression of KRAS^G12C^ leads to the promotion of translation rates in an mTORC1/p70S6K-independent mode, which is mediated mainly by the hypophosphorylation of eIF2α. Our results indicate that KRAS^G12C^ oncogenic signaling can differentially target translation initiation in different oncogenic contexts and provides details on the effects of KRAS^G12C^ signaling on translation initiation. Targeting the translation machinery in KRAS^G12C^-driven lung adenocarcinomas could possibly improve the very promising ongoing attempts to specifically target KRAS^G12C^. 

## Data Availability

Not applicable.

## Supplementary Materials

The following are available online at https://www.mdpi.com/1422-0067/22/4/2222/s1.
