# Supplementary material for: KRASG12C Can Either Promote or Impair Cap-Dependent Translation in Two Different Lung Adenocarcinoma Cell Lines
Source: Int J Mol Sci. 2021 Feb 23;22(4):2222. doi: 10.3390/ijms22042222 (PMC7926983; doi:10.3390/ijms22042222)
Supplement: Supplementary file 1 [file ijms-22-02222-s001.pdf]

International Journal of *Molecular Sciences*

MDPI

Supplementary Materials: KRAS^{G12C} Can Either Promote or Impair Cap-Dependent Translation in Two Different Lung Adenocarcinoma Cell Lines

George Kyriakopoulos, Vicky Katopodi, Ilias Skeparnias, Eleni G. Kaliatsi, Katerina Grafanaki and Constantinos Stathopoulos

Supplementary Figures

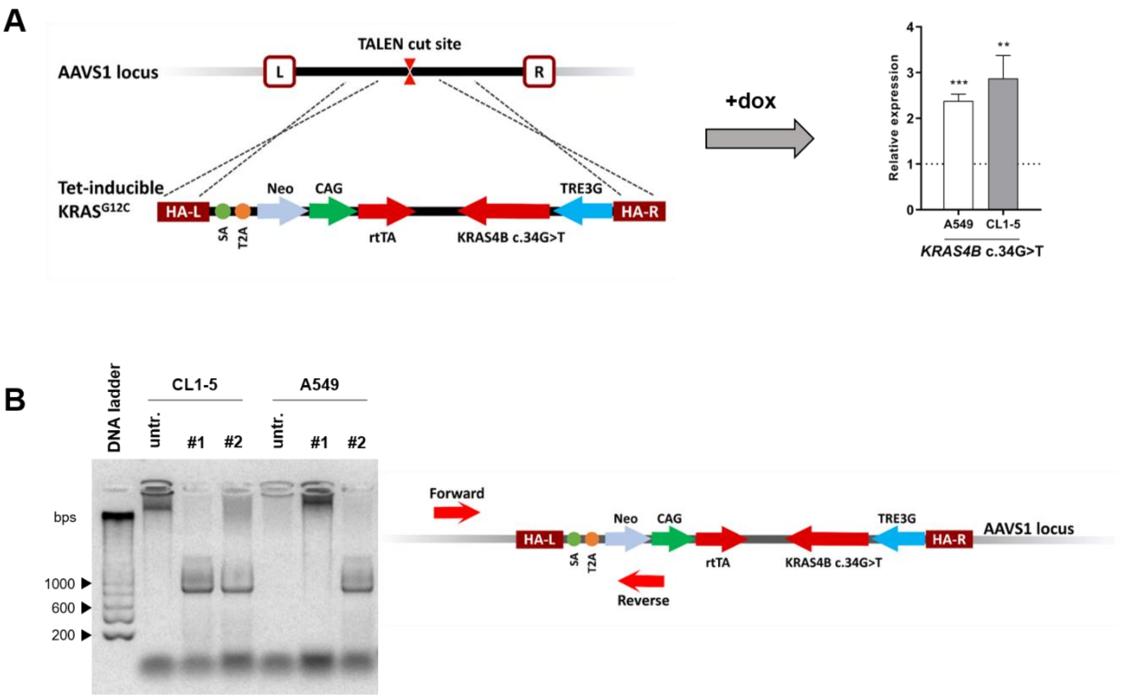

Figure S1. Generation of A549 and CL1-5 KRAS^{G12C} Tet-inducible stable cell lines. (**A**) TALE Nucleases Left and Right are designed to induce a double-strand brake at a specific point on the AAVS1 locus of the human genome. Homologous recombination with the homology arms of the construct containing the TRE3G promoter and the KRAS4B c.34G>T gene sequence were incorporated into the specific locus. After clonal selection, addition of doxycycline in the chosen clones' medium led to almost 3-fold upregulation of KRAS4B c.34G>T gene. The expression levels are shown as relative expression compared to the levels of endogenous *KRAS* gene of the control cells (dotted line) using the $2^{-\Delta\Delta Ct}$ method. Data are compared by using unpaired Student's t-test and are presented as means±SD of biological triplicates. *P*-values are indicated with *$p < 0.05$; **$p < 0.01$; ***$p < 0.001$. (**B**) Confirmation of the selected clonal cell cultures from A549 and CL1-5 cells by PCR using a set of primers that amplifies the expected 1033bps PCR product only if the construct is integrated at the AAVS1 locus. In CL1-5 cells both clones were positive and clone #1 was selected and in A549 cells clone #2 was positive.

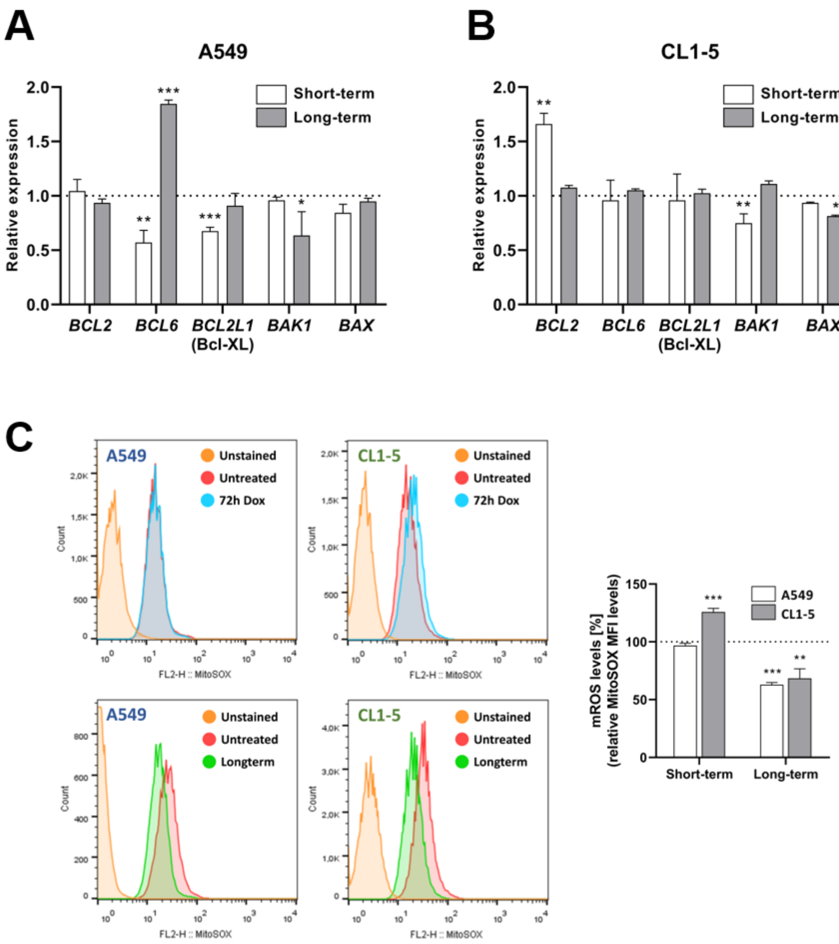

Figure S2. Expression levels of BCL2 family members and mitochondrial ROS production. (**A and B**) The mRNA levels of key members of the BCL2 family were measured by RT-qPCR after short-term (72 h) and long-term (>1 month) induction of KRAS^{G12C} in A549 and CL1-5 cells. All levels are shown as relative expression compared to the control (dotted line) using the $2^{-\Delta\Delta Ct}$ method. Data are compared by using unpaired Student's t-test and are presented as means±SD of biological triplicates. P-values are indicated with *$p < 0.05$; **$p < 0.01$; ***$p < 0.001$. (**C**) Mitochondrial ROS production were measured by MitoSOX staining and flow cytometry after short-term (72 h) and long-term (>1 month) induction of KRAS^{G12C} in A549 and CL1-5 cells. ROS levels are shown as relative (%) MFI levels upon MitoSOX staining compared to the control (dotted line). Data are compared by using unpaired Student's t-test and are presented as means±SD of biological triplicates. *P*-values are indicated with *$p < 0.05$; **$p < 0.01$; ***$p < 0.001$.

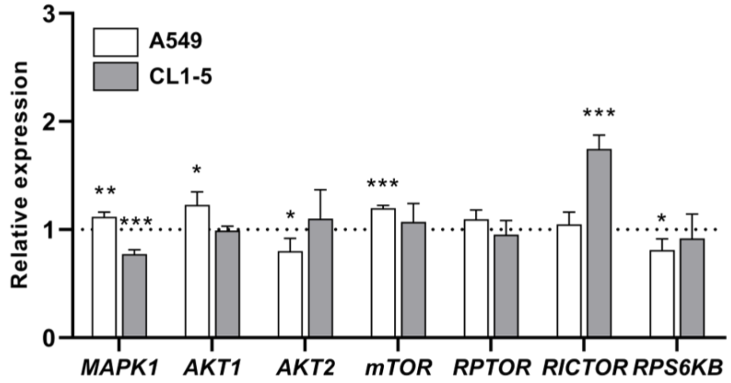

Figure S3. mRNA levels of key components of MAPK and PI3K/AKT/mTOR signaling pathways. Expression levels of genes involved in MAPK and PI3K/AKT/mTOR signaling pathways after induction of KRAS^{G12C} in A549 and CL1-5 cells, as identified by RT-qPCR. All levels are shown as relative expression compared to the control (dotted line) using the $2^{-\Delta\Delta Ct}$ method. Data are compared by using unpaired Student's t-test and are presented as means±SD of biological triplicates. *P*-values are indicated with **p* < 0.05; ***p* < 0.01; ****p* < 0.001.

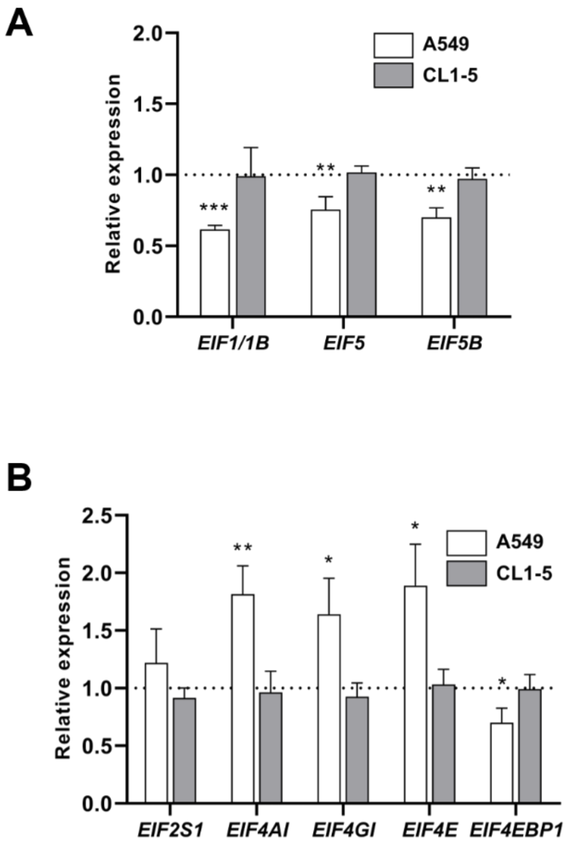

Figure S4. Expression profile of genes involved in the fidelity of start codon selection and cap-dependent translation. (**A**) The mRNA levels of key components involved in the fidelity of mRNA scanning were measured by RT-qPCR after induction of KRAS^{G12C} in A549 and CL1-5 cells. (**B**) The expression profile of *EIF2S1* gene and the genes coding for members of the eIF4F complex was identified by RT-qPCR after induction of KRAS^{G12C} in A549 and CL1-5 cells. All levels are shown as relative

expression compared to the control (dotted line) using the $2^{-\Delta\Delta Ct}$ method. Data are compared by using unpaired Student's t-test and are presented as means±SD of biological triplicates. *P*-values are indicated with $*p < 0.05$; $**p < 0.01$; $***p < 0.001$.

Supplementary Tables

Table 1. List of antibodies used for Western blot analysis.

Antibody	RRID	Catalog No.	Clone	Company
β-Actin	AB_330288	4967	polyclonal	Cell Signaling
phospho-ERK	AB_627545	sc-7383	E-4	Santa Cruz Biotech.
ERK 1/2	AB_2141283	sc-135900	MK1	Santa Cruz Biotech.
phospho-AKT (S473)	AB_329825	9271	polyclonal	Cell Signaling
phospho-mTOR (S2448)	AB_10691552	5536	D9C2	Cell Signaling
mTOR	AB_2105622	2983	7C10	Cell Signaling
RAPTOR	AB_2130791	sc-81537	10E10	Santa Cruz Biotech.
RICTOR	AB_10611167	sc-271081	H-11	Santa Cruz Biotech.
phospho-p70-S6K (T389)	AB_2269803	9234	108D2	Cell Signaling
p70-S6K	AB_331676	9202	polyclonal	Cell Signaling
IκB-α	AB_627772	sc-1643	H-4	Santa Cruz Biotech.
eIF1/1B	N/A	sc-390122	B-2	Santa Cruz Biotech.
phospho-eIF2α (S51)	AB_330951	9721	polyclonal	Cell Signaling
eIF2α	AB_2230924	9722	polyclonal	Cell Signaling
eIF5	AB_627507	sc-28309	E-10	Santa Cruz Biotech.
eIF5B	N/A	sc-393564	D-9	Santa Cruz Biotech.
phospho-eIF4E (S209)	AB_331677	9741	polyclonal	Cell Signaling
eIF4E	AB_2097675	2067	C46H6	Cell Signaling
phospho-4E-BP1 (S65)	AB_823413	9456	174A9	Cell Signaling
4E-BP1	AB_2097841	9644	53H11	Cell Signaling
c-MYC	AB_627266	sc-47694	9E11	Santa Cruz Biotech.
anti-puromycin	AB_2566826	MABE343	12D10	Millipore

Table S2. List of primers used for gene expression analysis.

Gene	Forward	Reverse
ACTB	AGCGAGCATCCCCCAAAGTT	GGGCACGAAGGCTCATCATT
BAK	TGCATTTGGCTGAATCAAGAACTT	AGTGGGAGAAGGACTATCAACACC
BAX-α	TCAGGATGCGTCCACCAAGAAGC	TGAGCACTCCCGCCACAAAGATG
BCL2	GCACTTCTCCCGCCGCTACCGC	AGGCCGCATGCTGGGGCCGIAC
BCL6	CATGCAGAGATGTGCCTCCACA	TCAGAGAAGCGGCAGTCACACT
BCL-XL	GGAAAGCGTAGACAAGGAGATGC	GGTGGGAGGGTAGAGTGGATGGT
KRAS4B	CAAGAGCGCCTTGACGATACA	CCAAGAGACAGGTTTCTCCATC
PTEN	GTGGAGTTTACCGGCAGCAT	CTGGCAGGTAGAAGGCAACT
c-MYC	TCCATGAGGAGACACCGCCCA	TGGGGCTGGTGCATTTTCGGT
MAPK1	CGCCGAAGCACCATTCAAG	AGAACACCGATGTCTGAGCA
AKT1	ATGCAGCATCGCTTCTTTGC	TGATCATCTGGGCCGTGAAC
AKT2	ACCACAGTCATCGAGAGGACC	GGAGCCACACTTGTAGTCCA
mTOR	AGCTGCCAAGTGCCGAGCAT	AGCAGCCATCCGGGCCATCTT
RPTOR	ATTCCCACCGCTCACTCATC	TTGACGCTCACACTCACGAT
RICTOR	TTTTCGGGGATTTCTGGATGC	GACAGCAAATGGGAAACCTCA
RPS6KB	TTGGTCAGGCAGTCAACACT	TGCAGGACACGTGGAGTAAC
EIF1/1B	TGTCCAAGGGATCGCTGATG	TTCAGTGAGCCACAAGCACT
EIF2S1	AATAGGCGCTTGACCCCAC	TGTTCTCTCCAGGGTTGTCG
EIF5	GCTCCGTATCCAGCAGTGAG	CATTCGACGCCTTTGAGCTT
EIF5B	GGGTGTTGAAGTGCCATCAAA	CCAATCATCCAATCCAGCATCC
EIF4AI	TATGACCTTCCCACCAACAGG	CCTCAGATGAGGTCAGCAACA

EIF4GI	TTTGAGACTCCCCTCCGAGT	CCTCGTCATACAGTGCGTCA
EIF4E	AGGACTGAAACCACCCTCCAGCA	TCGGGGGATTAGGAGTAGGGGTGG
EIF4EBP1	TCCTTGTGCCTCCACTGATG	CTAAGGAAGGGGTGGTTGCG

Table S3. List of primers used for miRNAs expression analysis.

Primers	Sequence
oligodT adapter	GCGAGCACAGAATTAATACGACTCACTATAGGTTTTTTTTTTTTVN
Outer primer	GCGAGCACAGAATTAATACGACT
miR-103	AGCAGCATTGTACAGGGCTATGA
miR-26a-5p	TTCAAGTAATCCAGGATAGGCT
miR-26b-5p	TTCAAGTAATTCAGGATAGGT
miR-92a-3p	TATTGCACTTGTCCCGGCCT
hsa-let-7a-5p	TGAGGTAGTAGGTTGTATAGTT
hsa-let-7b-5p	TGAGGTAGTAGGTTGTGTGGTT
hsa-let-7c-5p	TGAGGTAGTAGGTTGTATGGTT
hsa-let-7d-5p	AGAGGTAGTAGGTTGCATAGTT
hsa-let-7e-5p	TGAGGTAGGAGGTTGTATAGTT
hsa-let-7f-5p	TGAGGTAGTAGATTGTATAGTT
hsa-let-7g-5p	TGAGGTAGTAGTTTGTACAGTT
hsa-let-7i-5p	TGAGGTAGTAGTTTGTGCTGTT
